# Laser-Induced Selective Electroless Plating on PC/ABS Polymer: Minimisation of Thermal Effects for Supreme Processing Speed

**DOI:** 10.3390/polym12102427

**Published:** 2020-10-21

**Authors:** Karolis Ratautas, Vytautas Vosylius, Aldona Jagminienė, Ina Stankevičienė, Eugenijus Norkus, Gediminas Račiukaitis

**Affiliations:** Center for Physical Sciences and Technology, Savanoriu Ave. 231, LT-02300 Vilnius, Lithuania; vytautas.vosylius@ftmc.lt (V.V.); aldona.jagminiene@ftmc.lt (A.J.); ina.stankeviciene@ftmc.lt (I.S.); eugenijus.norkus@ftmc.lt (E.N.); gediminas.raciukaitis@ftmc.lt (G.R.)

**Keywords:** laser treatment, selective electroless platting, heat accumulation

## Abstract

The selective surface activation induced by laser (SSAIL) for electroless copper deposition on Polycarbonate/Acrylonitrile Butadiene Styrene (PC/ABS) blend is one of the promising techniques of electric circuit formation on free-shape dielectric surfaces, which broadens capabilities of 3D microscopic integrated devices (3D-MIDs). The process consists of laser excitation, chemical activation of laser-excited areas by dipping in a liquid and electroless copper deposition of the laser-treated areas. The limiting factor in increasing throughput of the technology is a laser activation step. Laser writing is performed by modern galvanometric scanners which reach the scanning speed of several meters per second. However, adverse thermal effects on PC/ABS polymer surface abridge the high-speed laser writing. In this work, an investigation was conducted on how these thermal effects limit surface activation for selective metal deposition from the view of physics and chemistry. An advanced laser beam scanning technique of interlacing with precise accuracy and the pulse-on-demand technique was applied to overcome mentioned problems for fast laser writing. Initially, the modelling of transient heat conduction was performed. The results revealed a significant reduction in heat accumulation. Applied methods of laser writing allowed the overall processing rate to increase by up to 2.4 times. Surface morphology was investigated by a scanning electron microscope. Energy-dispersive X-ray spectroscopy was used to investigate the modification of atomic concentration on the surface after laser treatment. Experiments did not show a correlation between surface morphology and electroless plating on laser-treated areas. However, significant variation in the composition of the material was revealed depending on the surface activity for electroless plating.

## 1. Introduction

A microscopic integrated device (MID) is an injection-moulded thermoplastic part with electronic circuits directly integrated on a polymeric component. It offers material, weight and cost savings by the elimination of connectors between separate printed circuit boards (PCBs), the shortening of the process chain and integration of contact surfaces, e.g., for switches, sensors and antennas [1]. MIDs have great potential in automotive, aviation, lighting, computing or even medical sectors where emerging innovation requires the increase of the number of the electronic components in a device. The MID approach requires a combination of various technologies to fabricate a product from different materials. A final product integrates multiple mechanical and electrical functionalities.

The main technological problem of MIDs is that of producing electrical circuit traces on plastic parts. Standard known techniques such as photolithography cannot be applied conventionally since the parts generally have complex 3D geometrical shapes. There are several methods of the laser-induced local metal deposition on polymers than can be used for circuit trace fabrication: metal nano-ink printing [2,3], ink paste layer sintering [4,5], laser-induced selective activation [6] and laser direct structuring (LDS) [7]. Injection printing uses liquid ink, usually made from silver nanoparticles. The thermal or piezoelectric method can be applied for droplet ejection. Presently, almost all industrial machines use piezoelectric heads [2]. There are several methods for ink paste sintering, but all of them have many similar procedures. A nonconductive ink solution based on metal compounds is reduced to metal atoms by laser irradiation. Kang et al. [4] made ink paste by dispersing copper oxide (CuO) particles into a reducing agent solution. The solution consisting of polyvinylpyrrolidone (PVP *M*_w_ 10,000, Aldrich, 13 wt %) and ethylene glycol (Aldrich, 27 wt %) was mixed with nanoparticles by an ultrasonic wave. Finally, the CuO NP solution with a viscosity of 5000 cps was achieved [4]. The solution was deposited on polyimide (PI) by a spin-coating technique. Ytterbium-doped fibre laser was used for irradiation of the deposited layer. Both pulsed nanosecond and CW lasers were applied. Photon energy of the irradiating laser had an energy high enough to break Cu–O bonds. After bond breaking, a reduction of Cu ions by ethylene glycol took place. The results were checked by energy-dispersive X-ray spectroscopy (EDS), and it was found that 90% of Cu ions could be reduced by pulse laser irradiation.

In a study carried out by Chen et al. [5], PI films were treated by KOH solution. As a result, PI became hydrolysed, and potassium polyamine was generated. Later, the specimen was immersed in the AgNO_3_ solution, allowing potassium ions to be exchange by the Ag ions. Laser writing was performed by Nd:YAG laser nanosecond laser with the IV harmonic wavelength. After laser irradiation, the Ag ions were reduced to Ag^0^ [5]. The disadvantages of both processes are very similar concerning the difficulties of thin layer deposition. In the case of the complex 3D surface, it becomes almost impossible.

Another technological problem is very low laser writing speed in the hundreds of microns/s during the reduction process. Zhang et al. presented a technology [6] for selective copper plating on laser-modified areas in a water environment, followed by chemical palladium activation and electroless plating. Firstly, the polymeric specimen—polycarbonate—was immersed in distilled water. The fundamental radiation of an Nd:YAG nanosecond laser was used to modify the polymer surface in the water. After the treatment, a sample was activated in palladium colloidal solution for several minutes. The rinsing after activation with palladium was performed in the distilled water. The last step, electroless copper plating, was applied for deposition of a conductive circuit. The selective plating process was also been attempted by performing laser activation in the air. However, no selective plating was achieved. The last two methods utilise chemical metal deposition in addition to laser treatment. All of them except LDS face difficulties when they are applied to three-dimensional parts.

LDS is the method of using precursors mixed in a polymer matrix [7]. These precursor additives are activated during the laser writing process by converting them into a catalyst for electroless deposition of the metal. Thus, the laser-treated area can be selectively plated [7]. LDS is a state-of-the-art method and the most commercialised process for MID production. There are a few commercial materials for LDS available on the market. However, most of the LDS polymers are based on expensive metal–organic fillers, such as palladium-based metal–organic compounds or microparticles of copper oxide spinel crystal [7], which increases the price of the raw material several times (for example, a cost ratio is 1:4 in the case of comparing pure PC/ABS to one with LDS additives) [8]. Therefore, the cost of the material limits the further expansion of the technology to the automotive and consumer goods sectors. In addition, a high required concentration of metal–organic additives reduces mechanical properties of a moulded part—moreover, metal-based additives in the polymer matrix shield electromagnetic radiation and limit MID application in the gigahertz frequency range [9,10].

One of the most promising technologies to overcome the aforementioned challenges is the selective surface activation induced by laser (SSAIL) [11,12]. The process includes laser modification of the polymer surface with a short-pulse laser, chemical activation of laser-modified areas and electroless metal deposition on the locally activated surface. An essential parameter for industrial application is the processing speed. The SSAIL technology consists of three chemical steps of dipping into liquids and one laser treatment step. From an industrial point of view, the chemical process could be easily upscaled by increasing the size of baths, allowing a large number of parts to be processed at once [13]. The laser process should be fast as laser time is expensive [8]. SSAIL technology offers high laser modification speed for high-performance polymeric materials like PolyEther Ether Ketone (PEEK) and Liquid Crystal Polymer (LCP) [12]. However, the rate of laser processing of widely used engineering plastics such as PC, ABS or PC/ABS is much lower. For example, for one of the most popular automotive engineering plastics, ABS, the laser writing speed of the SSAIL process is only 0.5 m/s [14]. The higher speed is limited by the unwanted thermal effects that can be caused by heat accumulation [15]. Thermal effects can result in the reduced adhesion to a substrate or lower spatial resolution of plating, or they can lead to areas not being covered by metal at all. There are several known solutions to the problem, such as cooling by gas agitation [16] or by water [17]. However, these methods are applied and then processing is carried out through a nozzle while the sample is positioned only by the mechanical axis. In the SSAIL process, fast laser processing is achieved by galvanometric scanning, and implementation of these cooling techniques would be complicated or even impossible. Another option for the elimination of thermal effects is to apply an advanced laser writing technique. J. Martan et al. have shown a special shifted laser writing method for high-speed texturing of metals [18,19] with a polygon scanner. Their approach permitted the processing speed to be significantly increased.

In this work, the laser writing technique at the maximum scanning speed using interlacing of pulses and lines with overlapping on the next scanning runs over the same area was implemented for the SSAIL process on PC/ABS polymer substrate. The potential benefits of the new technique were initially checked by finite element method (FEM) modelling of the heat equation after laser beam absorption in PC/ABS. The experiments were carried out using various laser processing parameters for the advanced laser writing method. The quality of the final plated copper layer was checked to determine the impact of the laser scanning approach. Optical and SEM microscopy, electrical sheet resistance and energy-dispersive X-ray spectroscopy (EDS) were applied to check the quality of the deposited copper layer.

## 2. Materials and Methods

### 2.1. Selective Deposition Using SSAIL

**Figure 1:**
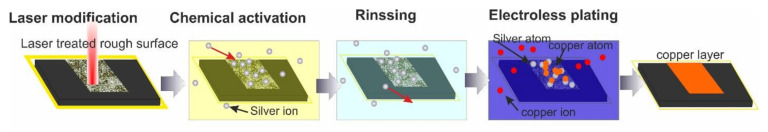
Steps of selective surface activation induced by laser (SSAIL) process: laser modification, chemical activation by dipping in the aqueous solution of silver nitrate, rinsing in distilled water and electroless copper deposition in the bath.

Plaques with the thickness of 3 mm, produced from PC/ABS compound (LNP Thermocomp compound NX10302; SABIC, Riyadh, Saudi Arabia) by injection moulding, were used as a substrate for selective copper deposition. The Nd:YVO_4_ picosecond laser Atlantic (Ekspla) with the pulse duration of 10 ps, pulse repetition rate up to 1 MHz and max average power up to 60 W was used for surface modification of the polymer. The laser beam had the Gaussian intensity distribution. Beam translation over a sample surface was performed by galvanometric scanner excelliSCAN (Scanlab GmbH, Puchheim, Germany). After the laser treatment, PC/ABS samples were washed with 99.8% ethanol (Sigma-Aldrich, Saint Louis, MO, USA) and rinsed with distilled water afterwards. For chemical activation, a highly diluted silver nitrate (Sigma-Aldrich, Saint Louis, MO, USA) solution (~10^−5^ M) was used. The activation step was followed by rinsing in distilled water for 1 min. Finally, the electroless copper deposition was performed for 40 min at 30 °C. The copper plating bath contained copper (II) sulphate pentahydrate (0.12 M), formaldehyde (0.3 M), sodium hydroxide (1.2 M), sodium carbonate (0.3 M) and sodium potassium tartrate (0.35) (all from (Sigma-Aldrich, Saint Louis, MO, USA) and had a pH of 12.7. All steps of the SSAIL method are shown in Figure 1.

### 2.2. Laser Writing Technique

**Figure 2:**
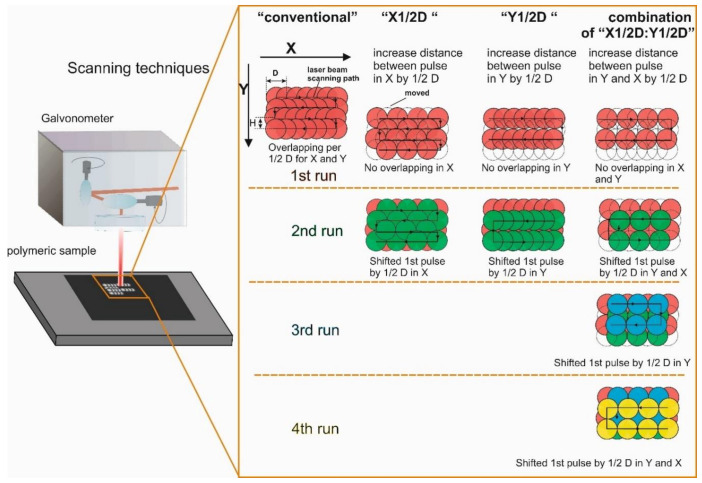
Laser writing techniques using a galvanometer scanner. D is the diameter of the laser pulse, and H is the hatch (the distance between laser-written lines). The 1st, 2nd, 3rd and 4th runs refer to the number of laser scans over the area. Arrows in the figure indicate the scanning direction. The X-axis is the direction of laser writing.

Laser writing was done by scanning rectangular areas with the size of 2 × 10 mm^2^ and 0.15 × 10 mm^2^. Laser pulses were overlapped by 50% of the beam diameter (beam diameter was 30 µm at 1/e level) in both X and Y directions, i.e., writing and adjacent scanned lines directions, as shown in Figure 2. Conventional scanning was performed by writing parallel partly overlapping lines, as shown in Figure 2 under “conventional”. The conventional scanning experiment was executed as a reference for comparison. The interlacing pulse scanning procedure was applied by increasing the distance between the pulses in X (scanned line direction) or Y directions (hatch between scan lines) to have non-overlapping pulses in one or both directions during the particular run over the scanned area. Overlapping of pulses was performed during subsequent runs of the scan by shifting the first pulse by 1/2D, as shown in Figure 2. The total irradiation dose was the same for each writing technique, in comparison with the conventional scanning. The first type of scanning was performed by increasing the distance between pulses in the X direction by one-half of the beam diameter, “X1/2D”, and the overlap of pulses in the X direction was achieved during the second run, as shown in Figure 2. The second type of laser beam translation was done by increasing the distance between pulses in the Y direction by one-half of the beam diameter, “Y1/2D”, and the overlap of pulses in the Y direction was achieved during the second run, as shown in Figure 2. The third type of scanning was a combination of scanning type 1 and scanning type 2, where the distance between pulses was increased in the Y and X directions by one-half of the beam diameter, “X1/2D:Y1/2D”, and overlap of pulses was achieved during the second, third and fourth runs as shown in Figure 2. Moreover, regimes where the distance between pulses more than 1/2D were examined as well, namely X1D and Y1D (by the diameter of the laser spot in the X or Y direction); X1D:Y1D (combination thereof); and X3/2D, Y3/2D and XY3/D (by 3/2 of a diameter of the laser spot in X or Y direction or both).

A single pulse-on-demand technique was used to synchronise the position and timing of the laser pulse over the scanned area during each subsequent scanning run. The accuracy of the position was higher than 2 µm.

### 2.3. Analysis and Measurements

A scanning electron microscope (JSM-6490LV; JEOL ltd., Tokyo, Japan) with EDS and an optical microscope (Olympus BX52, Olympus, Tokyo, Japan) with a digital camera were used to analyse the surface after the laser irradiation and metallisation steps. The optical microscope contained objectives in the range of 5–50 × magnification. Bright and dark field modes were used. Before the SEM analysis, samples were coated with 50 nm gold layer using the magnetron sputter coater Q150T ES (Quorum Technologies Ltd., Lewes, UK) to avoid charging of polymer surface. Electrical sheet resistance measurements were performed using the four-probe technique [12].

### 2.4. Modelling of the Heat Conduction after Laser Pulse Exposition to a Substrate

Simulation of temperature dynamics after laser irradiation was carried out using COMSOL Multiphysics software. Transient temperature distribution in PC/ABS substrate was simulated using the pulsed laser irradiation with the 532 nm wavelength and the Gaussian beam profile. Finite element method was used to solve the transient heat conduction (Equation (1)) [20]. The 3D model was built for the Gaussian laser beam heat source (Equation (2)) [21], assuming that 80% of laser energy was absorbed by linear absorption. The heat source was applied for four pulses scanned in the line by conventional scanning and scanning by interlacing pulse method using several runs to get overlapping, where the distance between pulses on the first run was increased by 1D, in comparison with the conventional scanning: X1D. The third pulse was applied after a single run time, and the fourth pulse was applied after the time of another single run, as shown in Figure 4.

(1) $$\rho C\frac{\partial T}{\partial t}=\nabla \left(\kappa \nabla T\right)+Q$$

Here, ρ is the material density (1.25 g/cm^2^ for PC/ABS), κ is the thermal conductivity (0.25 W/m^2^ for PC/ABS), Q is the heat source (described by Equation (2) [21]), T is the temperature, C is the thermal heat capacity and t is time.

(2) $$Q_{n}=\frac{P}{f\times t_{p}\times \omega_{0}{}^{2}\times \mathrm{pi}}\times \alpha \times \left(1-R\right)\times \exp{\left(-\frac{x-x_{n}}{\omega_{0}}\right)}^{2}\times \exp{\left(-\frac{y-y_{n}}{\omega_{0}}\right)}^{2}\;\times \exp\left(-\alpha \times z\right)\times \mathrm{rec}1\left(t/t_{p}\left[\frac{1}{s}\right]\right)$$

**Figure 3:**
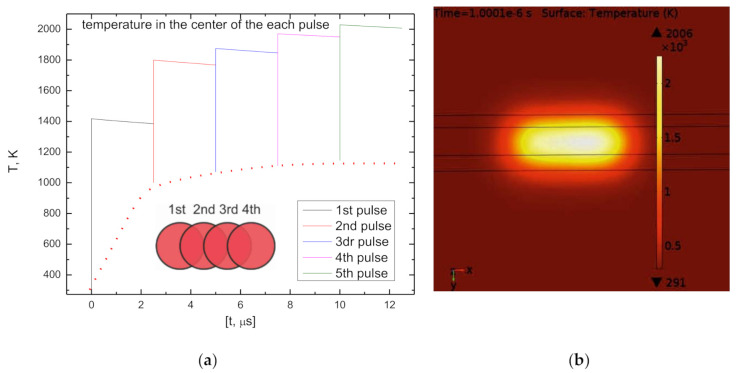
The transient temperature model for conventional scanning: (**a**) in the centre of the pulse; (**b**) the temperature distribution on the surface after 4th pulse.

Here, α is the absorption coefficient of PC/ABS blend, P is the laser power, x_n_ and y_n_ are the shifts of the coordinate depending on the subsequent position of the pulse, ω_0_ is the laser beam radius (at energy level 1/e^2^, it was 24 µm), z is the coordinate in the laser beam propagation direction, t_p_ is the pulse duration (10 ps), rec1(t) is the rectangular function to turn off the heat source and n refers to the pulse number. The modelling was performed for the 10 W average power of laser irradiation working at 400 kHz pulse repetition rate, and the highest scanning speed (4.8 m/s) was chosen for the model. The time interval between pulses was 2.5 µs, and the interval of one scanning run over the rectangular area of 2 × 10 mm^2^ was 0.25 s (in the case of X1D approach). Figure 3 presents the transient temperature variation in the middle of the pulse at the surface point when the conventional scanning technique was applied for five pulses. Temperature accumulation was observed up to 1000 K before saturation. Figure 3b shows the distribution of surface temperature right after the fourth pulse, and the heat-affected scanned path can be seen.

**Figure 4:**
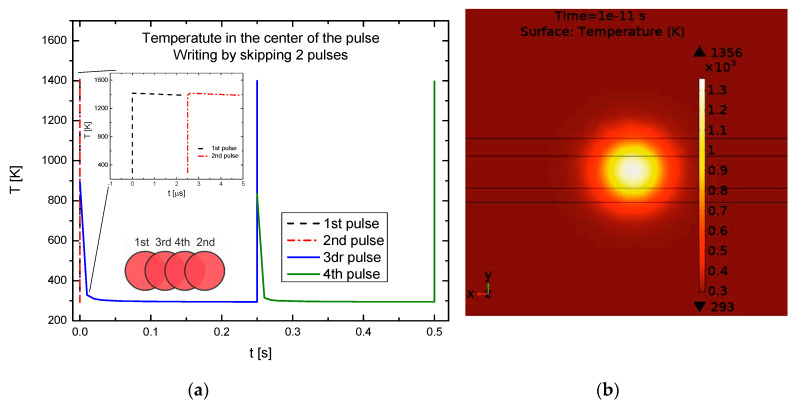
The transient temperature model for scanning type of X1D: (**a**) in the centre of the pulse; (**b**) the temperature distribution in K on the surface after 4th pulse.

The elimination of heat accumulation can be observed in Figure 4a, where the transient variation of temperature on the surface in the centre of the pulse is shown after the fourth pulse when the distance between pulses in the first run was increased by 1D in the X (laser writing) direction, and overlapping of pulses was achieved by the second and third runs. It can be seen that the maximum temperature increases up to almost 1400 K and then decreases to almost the initial temperature, and no accumulated heat remains until the next scan. Figure 4b presents temperature distribution right after the fourth pulse. No significant heat accumulation is observed.

## 3. Results

**Figure 5:**
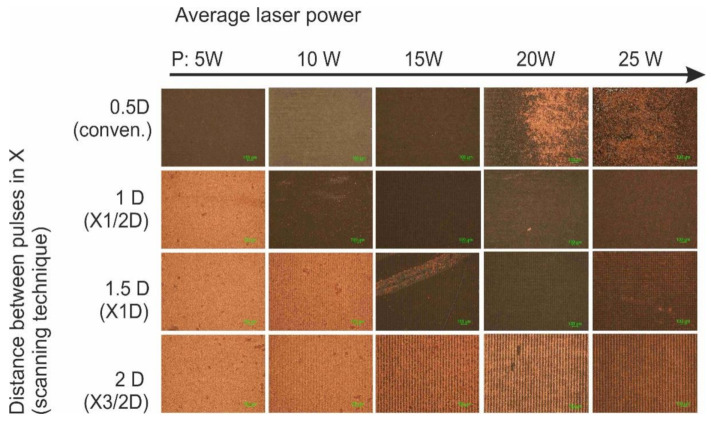
Optical microscope images of surface deposited with copper after electroless plating. Different average laser powers (5, 10, 15, 20 and 25 W (in columns)) and different types of scanning (conventional, X1/2D, X1D and X3/2D) were applied for comparison of laser excitation. The scale bar of all images corresponds to 100 µm.

Initially, the interlacing pulse technique was applied using X1/2D, X1D and X3/2D types of scanning with varying average laser power: 5, 10, 15, 20 and 25 W. Conventional scanning was applied as a reference. The maximum laser writing speed of 4.8 m/s was used at the 400 kHz pulse repetition rate. Optical microscope images, 1.5 × 1.5 mm^2^ in size, of surface areas after the complete SSAIL (final copper deposition) procedures for different laser writing techniques are shown in Figure 5.

Copper colour areas are clearly seen in Figure 5. The smooth plating over the entire treated surface was achieved, even after applying X1/2D type of scanning with only two scanning runs, with 5 W of average laser power. Increasing the distance between pulses in the X direction and the number of scanning runs led to a broader window of processing parameters for higher laser power and irradiation doses. This result clearly shows that laser processing speed for SSAIL process is limited by heat accumulation.

**Figure 6:**
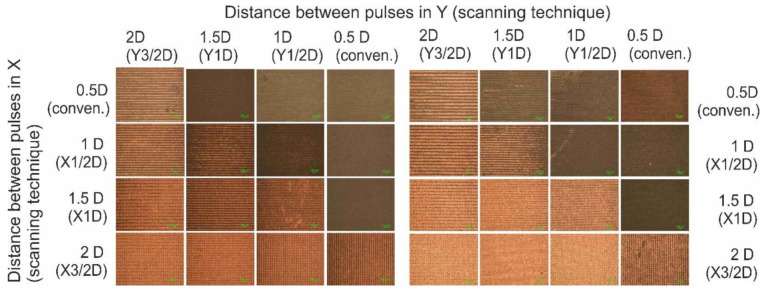
Optical microscope images of surface deposited with copper after electroless plating. The different types of interlacing pulse writing were applied: X1/2D; X1D, X3/2D, Y1/2D (in lines); Y1/2D, Y1D, Y3/2D (in columns); and a combination of them at the cross-sections. The average laser power was (**a**) 15 W and (**b**) 25 W at 400 kHz. The scale bar of all images corresponds to 100 µm.

SSAIL, a selective copper deposition procedure, was applied for interlacing pulse technique by increasing the distance between pulses in the Y direction, in other words, increasing the distance between parallel scanned lines, as explained in Figure 2. The Y1/2D, Y1D, Y3/2D types of laser writing were performed by scanning. When increasing the distance between pulses in the X direction, for example, by applying X1/2D writing, the processing time is doubled because the laser beam needs to travel over the whole scanned line in both scans. The same in the Y direction (perpendicular to scanned lines) does not increase processing time, since laser beam can simply skip a line within the short jump to the next line. The combination of both processes—increasing the distance between pulses in both directions—was tested as well. Optical images of deposited copper after applying different types of shifted pulse method are presented in Figure 6. Two different average laser power settings were used: 25 and 15 W. The laser power was chosen as shown in Figure 5, typically failing to obtain the plating if the conventional or the interlacing pulses in the X direction techniques were used. Applying only interlacing pulses in the Y direction did not allow continuous plating over the surface to be achieved, as shown in Figure 6, but the combination of both processes provided a fully covered surface for both values of average laser power. For higher average power (25 W), the combined process ensured better results, as shown in Figure 6b, as the Y1D × X3/2D area appears to be continuously covered.

**Figure 7:**
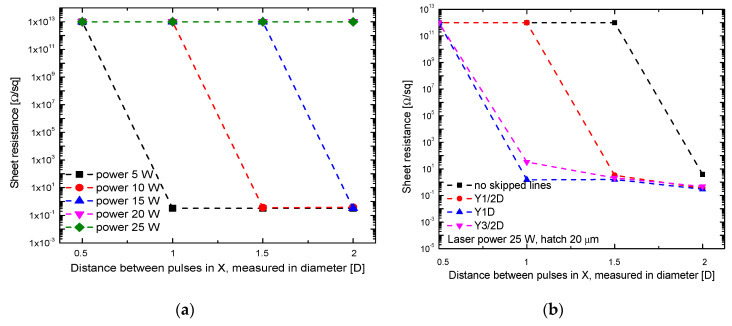
Dependence of the sheet resistance of deposited copper layer on the distance between pulses in the first run in the X direction (expressed in parts of diameter (D)) for various laser average power settings (**a**) and the different distance between pulses in the Y direction (**b**).

Electrical sheet resistance measurements were carried out to quantitatively evaluate the deposited copper layer. The four-probe technique was used for the resistance tests. Electrical measurements can indicate the quality of the plated surface even for optically undetectable problems, such as copper purity, or tiny areas of underplating. Results of the sheet resistance measurements are presented in Figure 7; they were obtained on the areas depicted in Figure 5 and Figure 6. It is clear from Figure 7a that the sheet resistance decreased after applying interlacing pulse scanning procedure in the X-direction. A drastic decrease in the sheet resistance indicates that a continuous copper layer is formed. For higher average laser power, a greater distance between pulses in the X direction is needed to achieve low sheet resistance values. In the case of 25 W, even after X3/2D type of scanning, the resistance remained high, showing that there was no continuous copper layer. After applying a combination of increasing the distance between pulses on the first run in both X and Y directions, it was possible to deposit a copper layer, even when the high laser power (25 W) was used, as demonstrated in Figure 7b.

**Figure 8:**
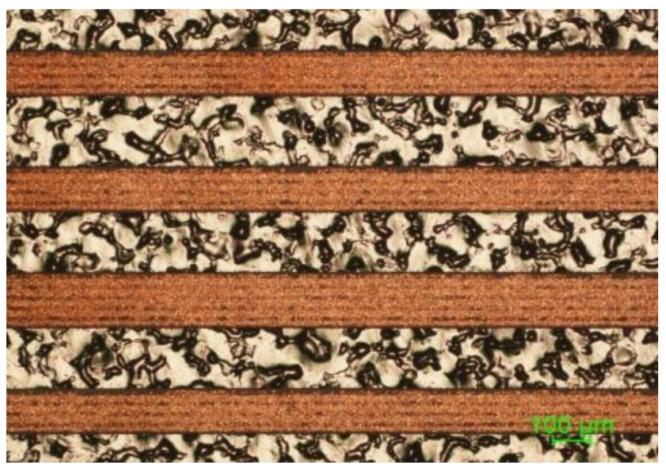
Copper lines after the SSAIL procedure when the Y3/2D scanning technique was applied. The average laser power was 5 W.

However, the application of interlacing pulses in the Y direction even after low power of irradiation did not guarantee the continuously plated surface when 4.8 m/s scanning speed was applied. Figure 8 shows the optical microscope image of deposited copper after application of 5 W average laser power and Y3/2D. In the picture, visible dark lines indicate that coverage is not full along the plated stripe.

**Figure 9:**
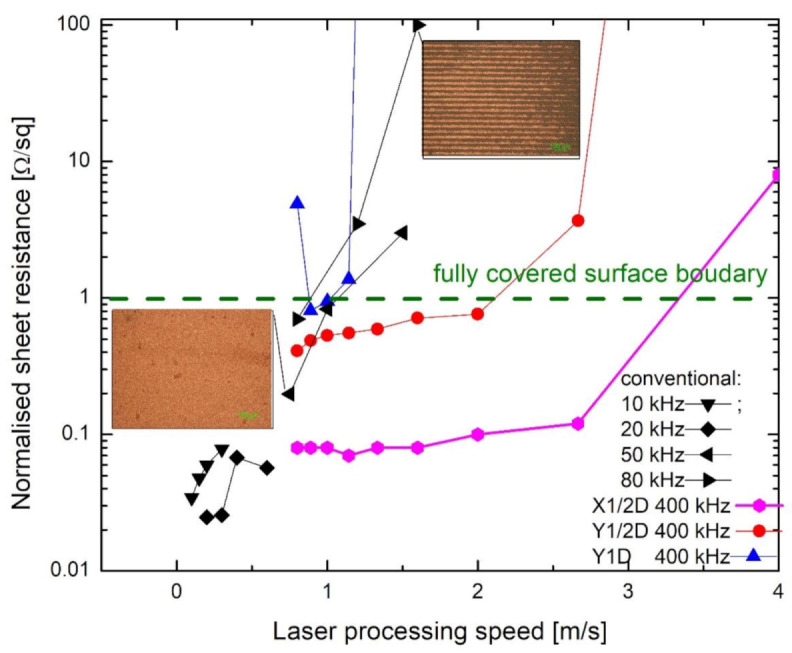
Dependence of the copper layer sheet resistance on laser processing speed for various average laser power settings (0.2 W at 10 kHz, 0.5 W at 20 kHz, 1 W at 50 kHz and 1.6 W at 80 kHz) and 5 W average laser power at 400 kHz with the X1/2D, Y1/2D and Y1D laser writing techniques. The scale bar of images corresponds to 100 µm.

Further samples were treated with at a lower speed of laser beam writing, 0.8–4.8 m/s for the X1/2D and Y1/2D, Y1D, Y3/2D scanning types, applying the 5 W average laser power and 400 kHz pulse repetition rate. The results were compared with the conventional scanning with several best possible regimes of laser irradiation and writing parameters [14]. The sheet resistance dependence on laser processing speed is presented in Figure 9. The results are described not by laser writing speed but by the overall processing speed instead, including that scanning by X1/2D requires two scans when compared with the conventional scanning with the same writing speed. The fully covered copper layer has the resistance of less than 1 Ω/sq, as shown in Figure 9 by the green dashed line. All values below this line were considered as continuously plated—the positive experimental result. The fastest processing speed for the conventional scanning technique was 1 m/s, and the value of resistance was very close to the boundary line. The processing speed could be increased if the Y1/2D scanning technique was applied. However, no improvement was observed when the Y1D technique was used.

The most favourable results were achieved for the X1/2D scanning technique. The process works with the highest 4.8 m/s laser writing speed and provides a 2.4 m/s processing speed. The best copper plating quality was achieved for the X1/2D scanning technique as well since the low sheet resistance of plated copper was measured. Lower sheet resistance in the case of conventional scanning at a low pulse repetition rate like 10 or 20 kHz could be caused by a thicker layer of copper, as the plating was carried longer [14].

**Figure 10:**
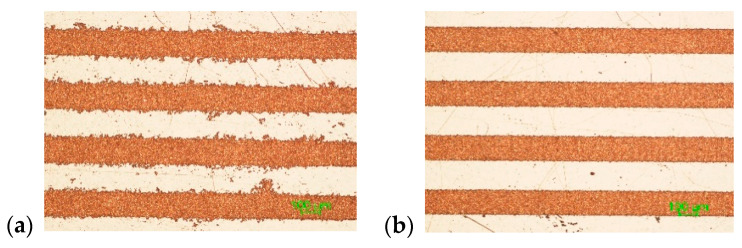

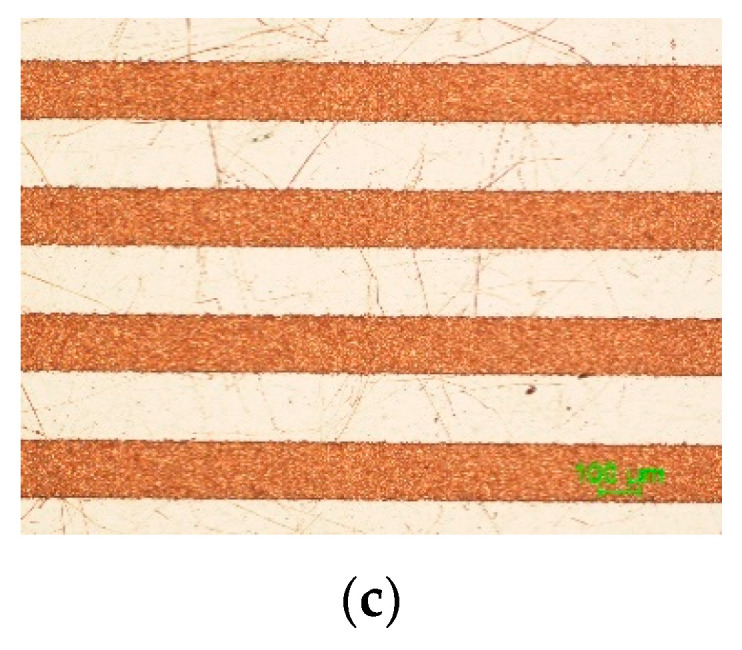
Plated lines after applying non-overlapped scanning in the first scanning run technique: (**a**) X1/2D; (**b**) X1D; (**c**) X3/2D. The scanning speed was 4.8 m/s, the pulse repetition rate was 400 kHz and the average laser power was 5 W. The scale bar of all three images corresponds to 100 µm.

Another critical parameter is plating selectivity. Figure 10 shows the plated lines with X1/2D, X1D and X3/2D scanning techniques using 5 W average laser power.

From Figure 10, it can be easily seen that the plated lines where X1D and X3/2D writing was applied have smoother edges. This could be a consequence of the mitigation of heat accumulation by interlaced scanning.

**Figure 11:**
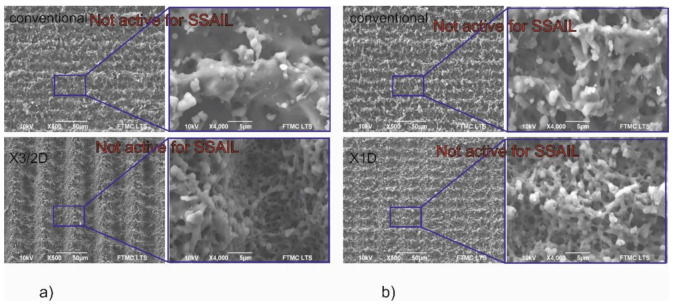
SEM image of PC/ABS surface treated with picosecond laser applying (**a**) 20 W and (**b**) 15 W average laser power. “Conventional”, “X3/2D” and “X1D” indicate the method of laser writing, as described in the experimental section.

Scanning electron microscope imaging was used to analyse the surface morphology. Laser-treated surfaces subjected to the interlacing pulses in the X direction writing technique were analysed and compared. In some cases, a significant decrease of surface melting was observed when the increase of the distance between pulses in the X direction was applied for the first scanning run. Figure 11 presents examples of surface morphology written by increasing the distance between pulses in the X direction and using conventional scanning. Figure 11a shows a surface irradiated with 20 W average laser power, and Figure 11b shows a surface irradiated with 15 W average laser power. When applying a greater distance between pulses on the first scanning run, the surface’s structure is entirely different—a submicron structure is formed. Meanwhile, after the conventional writing technique, the surface is remelted, and the structure has micrometre-sized features. Nevertheless, the significant reduction of thermal effects, like eliminating remelting of surface, in the modified surface in Figure 11a,b where X3/2D and X1D writing is applied is not suitable for copper deposition using SSAIL.

**Figure 12:**
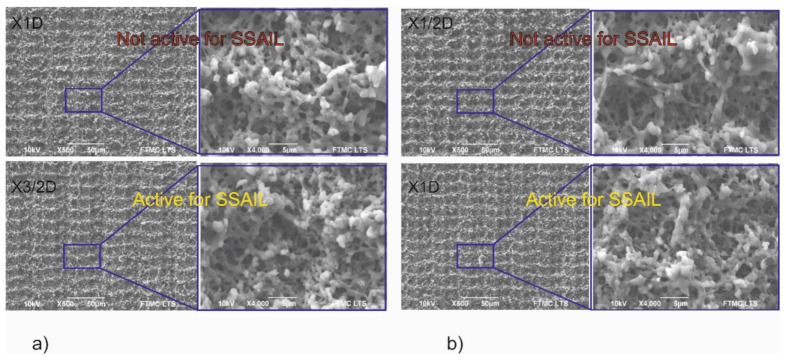
SEM image of PC/ABS surface processed with picosecond laser applying (**a**) 15 W and (**b**) 10 W average laser power. “X1D”, “X3/2D” and “X1/2D” indicate the method of laser writing, as described in the experimental section.

In Figure 12a, the surface processed with 15 W average laser power and scanned with X1D is compared with the surface treated with the same laser parameters but applying X3/2D writing technique. In Figure 12b, the surface processed with 10 W average laser power and scanned with X1/2D is compared with the surface processed with the same laser parameters but applying X1D writing. In both pictures, no significant difference can be seen as resulting after increasing the distance between pulses. However, the surface is not active for SSAIL, and no copper can be deposited after 15 W average laser power with X1D scanning treatment. The same could be said about the surface after 10 W laser power with X1/2D scanning treatment. However, after increasing the distance between pulses in the scanning direction by one-half of the beam diameter for both cases, the surface becomes active for SSAIL process. In other words, the chemical activation can take place in the irradiated areas. This result could be explained by reduced accumulation of temperature, although no morphological structure changes on the surface are observed. This result proves that SSAIL process is caused not only by the morphological structures on the surface after the laser modification.

All experimental investigations show that thermal accumulation is a limiting factor for fast laser writing in the SSAIL process. However, the consequences of the heat accumulation are not fully understood yet, since SEM pictures show that morphology can look almost identical for the active and non-active surfaces for electroless deposition using the SSAIL technique. Therefore, we assume that differences in surface activation could come from chemical modifications in the laser-treated areas.

**Figure 13:**
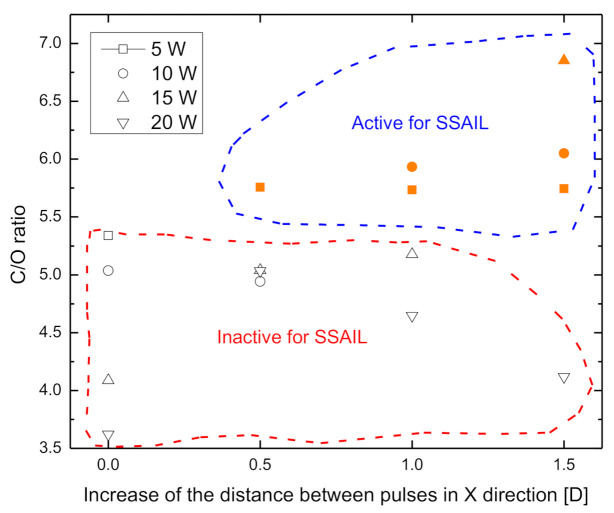
Dependence of the C/O ratio of atomic concentration on laser-modified PC/ABS surface on the distance between pulses in the X direction (measured as a part of pulse diameter) in scanning regime for the first scanning run. The measurements were done by EDS.

EDS was applied to measure the ratio of carbon to oxygen atomic concentration on the surface of polymer irradiated with the laser. Laser processing regimes indicated in Figure 5 were used for the treatment of samples. The C/O ratio dependence on the increase of distance between pulses for various average laser powers is presented in Figure 13. The reference ratio of atomic C/O concentration for the non-processed surface was 3.47. After the laser treatment, the C/O ratio increased, showing a decrease of oxygen content on the surface. Increase in the distance between pulses in the scanning direction resulted in a lower oxygen concentration when 5, 10 or 15 W average laser power was used. EDS measurements describe the relation between C/O ratio and activity of a surface for electroless copper deposition using the SSAIL process. Laser processing parameters when the surface of PC/ABS was activated for SSAIL are marked in orange colour in Figure 13. All surfaces which were active for SSAIL after the laser treatment had a C/O ratio above 5.75. This result shows that the decrease in oxygen content on the PC/ABS surface plays a crucial role in laser excitation process for chemical activation with silver nitrate.

**Figure 14:**
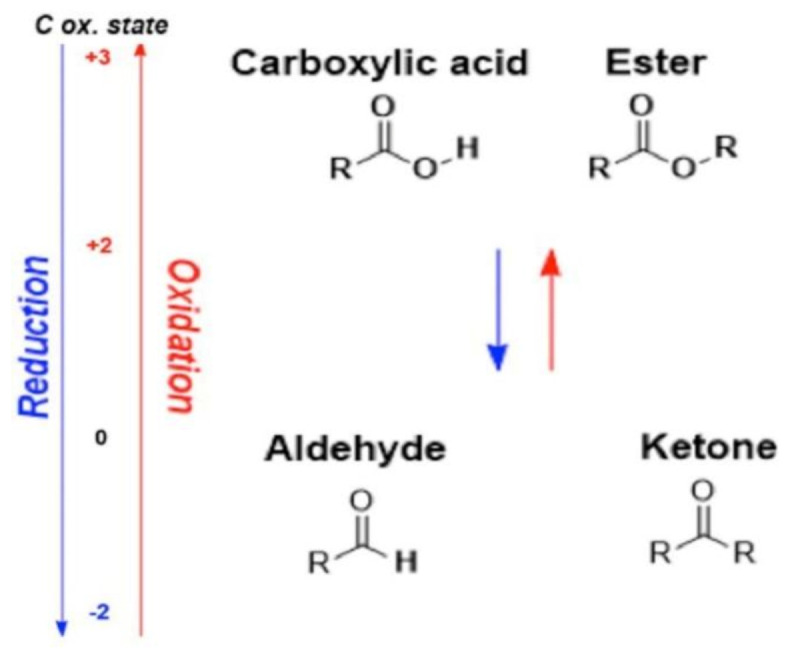
Scheme of the reduction of carboxylic and ester chemical groups to aldehydes and ketones.

There are some works that present the decrease in oxygen content on the polymer surface after laser irradiation with excimer lasers [22]. The excimer laser works in ultraviolet wavelength range of approximately 340–250 nm, while our research was performed with the 532 nm wavelength. Using an excimer laser, carbonyl and hydroxy groups (which contain oxygen) on polymer surface can be destroyed photochemically. Photon energy at the 532 nm wavelength is not high enough to break chemical bonds of hydroxy or oxygenated chemical groups. However, pulse duration (used in this work) is more than 1000 times shorter than that of excimer lasers, and nonlinear multiphoton absorption could be dominant, enabling chemical bonds to be broken with lower photon energy. Wu et al. [23] reported oxygenated chemical groups that were naturally formed on a polycarbonate surface in ambient air. The content of PC in PC/ABS blend is significantly higher: 70–90% by weight. Therefore, much more PC material interacts with laser irradiation than PC/ABS blend. Figure 14a shows the surface chemical condition of PC in ambient air. Carbonyl C=O groups are localised on the PC surface, and ester groups are also located very close to the surface. Polycarbonates can also contain some amounts of carboxylic groups. It is well known that during the reduction of esters or carbocyclic acids, ketones and aldehydes are formed, respectively. In both cases, the loss in oxygen amount was observed (see Figure 14).

**Figure 15:**
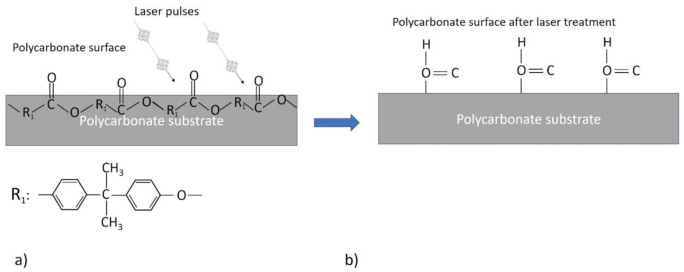
Schematic image showing the interaction of chemical groups on PC surface with laser pulses (**a**) and the broken bonds of carbonyl groups after laser treatment, leading to a formation of aldehyde groups on PC surface (**b**).

As the decrease of oxygen content on the surface was observed after the laser treatment for surface excitation, we can conclude that the picosecond laser pulse nonlinearly breaks bonds of carbonyl and ester groups directly on the surface or below it, and the reduction process takes place. As a result, the formation of the aldehydic groups on the surface could be expected, as shown in Figure 15b.

After the laser treatment, a sample was immersed in AgNO_3_ activation solution, and localised aldehydic groups on laser-treated areas reacted with Ag^+^ very easily and actively. As a result, metallic Ag was produced, which acted later as a catalyst for the electroless copper deposition process.

The suggested interpretation explains the selectiveness of plating for the SSAIL process. Moreover, it explains the different metal deposition behaviours on the surfaces that look almost identical regarding the surface morphology (Figure 12).

**Figure 16:**
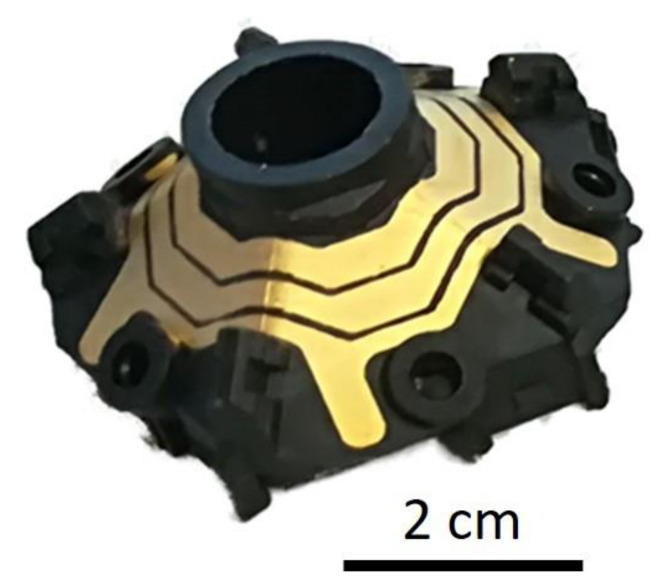
3D MID item after SSAIL procedure, using pulse interlacing technique.

SSAIL technology is orientated to practical industrial application. Therefore, the time for laser treatment of the real 3D items was estimated. Figure 16a presents a 3D MID part that was processed using pulse interlacing laser scanning method. The X1/2D writing technique was used for the machining of the item. In this example, the laser processing time for the conventional scanning method (1 min) was reduced to 25 s for a single item.

## 4. Conclusions

The use of the advanced laser scanning interlacing pulse technique, enabled by increasing the distance between pulses and obtaining overlapping during subsequent scanning runs by shifting a pulse in one-half of the laser spot diameter, showed possibilities to reduce thermal effects like heat accumulation on PC/ABS substrate for a high processing speed of 4.8 m/s. Heat conduction modelling of the PC/ABS surface after absorption of laser irradiation also revealed elimination of heat accumulation when the increase in distance between pulses in scanning direction for the first scanning run was done. The new tested laser writing techniques allowed increasing the laser processing speed of PC/ABS polymer for laser-induced selective electroless copper deposition by more than 2.4 times. Scanning electron microscope images testified that microscopic surface morphology appearing after laser modification of PC/ABS does not play the main role in the surface activity of selective electroless plating. However, the change in atomic concentration of the material varies depending on the surface activity of electroless plating, as a decrease in atomic oxygen content for increasing surface activity was observed. It was suggested that the picosecond laser pulse nonlinearly breaks the bonds of carbonyl and ester groups directly on the surface or below it, and the reduction process takes place. As a result, the aldehydic groups are formed on the surface. After the laser treatment, a sample was immersed in AgNO_3_ activation solution, and localised aldehydic groups on laser-treated areas reacted with Ag^+^ very easily and actively. This allows metallic Ag to be produced, which acts later as a catalyst for the electroless copper deposition process.
